# Molecular basis of lysosomal storage disorders in India

**DOI:** 10.1186/1755-8166-7-S1-P130

**Published:** 2014-01-21

**Authors:** Shweta P Kondurkar, Parag Tamhankar, Pratima Kondurkar, Ashwin Dalal, Jayesh Sheth

**Affiliations:** 1 ICMR Genetic Research Centre (NIRRH), Mumbai, India; 2 Center for DNA Fingerprinting and Diagnostics, Hyderabad, India; 3 Foundation for Research in Genetics and Endocrinology, Ahmedabad, Gujarat, India

## Background

Lysosomal Storage Diseases(LSD) are a group of rare recessive inherited metabolic disorders that result from the deficiency of a single enzyme required for the metabolism of lipids, glycoproteins or mucopolysaccharides. There is a lack of data on molecular basis of LSDs in India . The current study involves molecular analysis of patients with Tay Sachs disease(TSD) (*HEXA* gene), Sandhoff disease(SD) (*HEXB* gene), Gaucher disease (GD) (GBA gene), GM1 gangliosidosis (GG) (*GLB1* gene), metachromatic leukodystrophy (MLD) (*ARSA* gene), and Pompe disease (PD) (*GAA* gene).

## Materials and methods

Patients presenting during the year 2011-2013 with characteristic clinical features of the above LSDs underwent specific biochemical testing (leucocyte enzyme assay) followed by sequencing of the respective gene. Enzyme assays performed include total hexosaminidase and hexosaminidase B (TSD and SD), glucocerebrosidase (GD), beta galactosidase (GG), arylsulphatase A (MLD) and alpha glucosidase (PD). The molecular basis of disease in patients with enzyme deficiency was confirmed by bidirectional Sanger sequencing covering all the exons and exon-intron boundaries of the respective genes.

## Results

During the study period, 131 unrelated families across India were studied. These included 47 families of TSD, 36 families with SD, 15 families with GD, 8 families with GG, 15 families with MLD, and 10 families with PD. Ninety two families (70. 2 %) families showed consanguinity. The age range for patients was between 3 months to 7 years; however, one patient with PD was adult (21 years). The youngest patient had PD. The study identified 218 mutant alleles in 119 patients. Only ten alleles were recurrent. Founder mutation was identified in TSD patients from Gujarat (p.E462V) which will help screening in patients from this state. Also in SD, the mutation hotspot R284X represented 23.3 % of alleles (7/30). No mutations could be identified in 12 patients and the second mutation could not be identified in 10 patients, despite being biochemically confirmed.

## Conclusion

The study shows allelic heterogeneity in Indian patients with LSDs. A molecular screening strategy for the common mutations could be adopted for TSD and SD patients.

